# Neural Basis of Internal Attention in Adults with Pure and Comorbid ADHD

**DOI:** 10.1177/10870547221147546

**Published:** 2023-01-12

**Authors:** Halima Rafi, Ryan Murray, Farnaz Delavari, Nader Perroud, Patrik Vuilleumier, Martin Debbané, Camille Piguet

**Affiliations:** 1 University of Geneva, Switzerland; 2 University of Geneva, Campus Biotech, Switzerland; 3 Institute of Bioengineering, École Polytechnique Fédérale de Lausanne, Switzerland; 4 University Hospitals of Geneva, Switzerland; 5 University College London, UK

**Keywords:** adult ADHD, fMRI, comorbidity

## Abstract

**Objective:**

To examine whether putatively atypical neuronal activity during internal attention in ADHD yields insights into processes underlying emotion dysregulation.

**Methods:**

We used a word processing paradigm to assess neural activations in adults with ADHD (*N* = 46) compared to controls (*N* = 43). We measured effects of valence, applied partial-least squares correlation analysis to assess multivariate brainbehavior relationships and ran subgroup analyses to isolate results driven by pure ADHD (*N* = 18).

**Results:**

During internal attention, ADHD, compared to controls, have (1) increased activation in the right angular gyrus (rAG), which appears driven by pure, not comorbid, ADHD and (2) diminished activation in the insula and fronto-striatal circuitry. Diminished activations were driven by negatively-valenced internal attention and negatively correlated with increased affective lability within the ADHD group.

**Conclusion:**

Internal attention in ADHD is associated with increased rAG activation, possibly reflecting difficulty converging external and internal information, and diminished activation within emotion regulation circuitry.

## Introduction

Manifestations of attention-deficit/hyperactivity disorder (ADHD) evolve with age. In adults, ADHD primarily presents as difficulties in higher-order functions (Rösler et al., 2010; Wender, 1998), such as regulating attention, emotions, and enacting goal-directed behaviors (Kessler et al., 2005). Impairments in such interconnected domains can lead to long-term dysfunctional professional and interpersonal relationships (Biederman & Faraone, 2006; Faraone & Biederman, 2005), conveying a substantial lifetime burden on individuals with ADHD and society at large (Able et al., 2014). Questions about how specific brain regions and pathways are implicated in attention in ADHD can be researched through the broad lens of attention orientation, which can be defined as aligning attention with internal features of the self or with external sensory inputs (Posner, 1980). To date, few studies have examined differences in ADHD when attention is oriented inwards to sensations, emotions, and thoughts about the self (internal attention), in contrast to the more widely researched domain of outwardly oriented attention toward stimuli in the environment (external attention).

Internally oriented attention occupies much of our daily lives (Singer, 1966), and encompasses the higher-order function of self-mentalization. Self-mentalization is an interactive process between executive functions and imaginative capacities that allows us to attribute intentional mental states to ourselves and our behavior (Fonagy et al., 2002). The capacity to effectively self-mentalize is contingent upon adaptive attentional control and emotional self-regulation during early development. These abilities, in turn, allow for the self-governing necessary for adaptive social interactions (Barkley, 2015; Cortese et al., 2012; Durston et al., 2003). Emotional self-regulation is a core difficulty in ADHD (Barkley, 2015) and is closely associated to cognitive processes contingent on internal attention, such as interoception (Bud Craig, 2009; Critchley & Garfinkel, 2017; Tsakiris & Critchley, 2016). Interoception, the process of perceiving our body’s physiological state (Craig, 2002), is crucial for maintaining physiological equilibrium. It allows us to recognize and experience embodied emotions and recent research links higher interoception to an increased capacity for feeling bodily states and regulating emotions (Zamariola et al., 2019). When it comes to ADHD, existing literature is mixed with studies showing both preserved interoception (Kutscheidt et al., 2019) and impaired interoception in adults with ADHD. In short, it is likely that ADHD’s neurodevelopmental nature puts its population at risk for increased lifelong difficulties with various types of internal attention, like self-mentalization and interoception, but limited research exists on this topic (Perroud et al., 2017).

A rich literature on attention orientation in ADHD exists but most studies to date have focused on external, rather than internal, attention. Functional magnetic resonance imaging (fMRI) research on externally oriented attention shows a tendency in ADHD populations to be more distracted by salient stimuli (Forster & Lavie, 2016; Mason et al., 2005) and to have atypical activation in neural systems of executive functioning compared to healthy controls (HC) (Cortese et al., 2012). Brain regions consistently hypoactive in ADHD compared to HC during executive functions-centered tasks include the striatum (Durston et al., 2003), the ACC (Konrad et al., 2006), the PFC (Rubia et al., 2005; Vaidya et al., 1998), and the inferior frontal gyrus (IFG) (Durston et al., 2006; Rubia et al., 2005). Interestingly, in addition to altered perceptual processing, literature has posited that the driving force behind increased susceptibility to external distractors in ADHD is the tendency for them to be disproportionately focused on internal states (Castellanos et al., 2006; Van Den Driessche et al., 2017), leaving insufficient resources for attentional control. In terms of emotional effects, emotional external attention in ADHD has been well-studied, typically using temporal discounting and gambling tasks. Overall, studies show a tendency for ADHD populations to be hypersensitive to negatively valenced stimuli (López-Martín et al., 2013; Vetter et al., 2018; Wilbertz et al., 2017) and hyposensitive toward positively valenced stimuli (Conzelmann et al., 2009; Ibáñez et al., 2011). For example, a recent fMRI study showed that adolescents with ADHD had increased activation in the left AI and IFG during negatively valenced stimuli (Vetter et al., 2018). This finding was of special interest because insula is among the only brain regions with structural and functional abnormalities in children and adults with ADHD (Bud Craig, 2009; Norman et al., 2017). It is a hub in both the ventral attention (Carretié, 2014; Norman et al., 2017) and salience networks (Barrett & Satpute, 2013; Seeley et al., 2007), which are responsible for detecting and reorienting attention toward salient stimuli. The authors argued their findings support altered salience processing of negative emotional distractors in ADHD; notably, this is also a putative mechanism in other psychiatric disorders, such as anxiety and depression disorders (Gotlib & Joormann, 2010; Joormann et al., 2011).

Generally speaking, fMRI research on internal attention suggests it is modulated by brain areas associated with memory processes, self-generated thought, and affective processing; regions include the precuneus, anterior insula (AI) and pre-supplementary motor area (pre-SMA) (Wade-Bohleber et al., 2019) and subgenual and dorsal anterior cingulate cortex (ACC) (Schilbach et al., 2014). Internal attention such as self-related thinking is also associated with increased default mode network (DMN) activation (Andrews-Hanna et al., 2014). The DMN is a large-scale, functional brain network with hubs in the medial prefrontal cortex (mPFC) and the posterior cingulate cortex (PCC). It is typically activate when the mind is not directed toward a specific goal or object of thought (Andrews-Hanna et al., 2010; Takeuchi et al., 2011). The notion that this distinct neural network underlies the human sense of self has been the subject of considerable empirical research; neuroimaging studies using self-referential cognitive (Harrison et al., 2008) and mind-wandering tasks (Mason et al., 2007) show patterns of activation like those seen in resting-state DMN. However, when it comes to the intersection of internal attention and emotion, the neural correlates of these complex processes are understudied in ADHD. Given that emotional dysregulation is increasingly considered a core symptom of ADHD (Corbisiero et al., 2013; Retz et al., 2012), it is important to examine neural correlates of emotional internal attention. This line of research is relatively unexplored and may inform us on the pathways underlying difficulties with crucial abilities that pertain to the self, including both internal attention and emotional dysregulation in ADHD (Bateman & Fonagy, 2016; Perroud et al., 2017).

Importantly, ADHD shares high comorbidity rates with disorders such as major depressive disorder (up to 50% comorbidity rate (Angold et al., 1999)), anxiety disorders (up to 35% comorbidity rate (Busch et al., 2002; Jensen et al., 2001)) and externalizing disorders such as conduct disorder and oppositional defiance disorder (up to 50% comorbidity (Bird et al., 1988)). This poses a conundrum in scientific research, which is either conducted with “pure” ADHD samples, meaning patients with ADHD but without other diagnoses, or with samples having ADHD as well as other comorbid diagnoses. The first approach allows the isolation of behavioral and neural outcomes specific to ADHD, but at the cost of not being representative of ADHD in which comorbidities are the rule rather than the exception. The latter approach cannot disentangle which outcomes are related to comorbidities, and which are specific to ADHD itself.

The present fMRI study assessed patterns of brain activation related to internal attention and emotional valence in young adults with ADHD. Participants completed a word processing paradigm based on previously published paradigms (Davey et al., 2016; Kelley et al., 2002; Whitfield-Gabrieli et al., 2011) that required either internal or external attention to stimuli that were either positively or negatively valenced (for task details, please see Methods section 2.3.). Given previous fMRI literature on internal attention in HC as well as external attention in ADHD populations, we were especially interested in the brain activity of regions associated with self-processing, including the AI, operculum, IFG as well as regions consistently linked to emotion-processing such as the fronto-striatal circuitry. During internally oriented attention trials, we expected adults with ADHD compared to HC to show (a) decreased activation of self- and emotion-processing regions for positively valenced stimuli and (b) increased activation of self- and emotion-processing regions for negatively valenced stimuli. As a control measure, we also assessed brain activations to external positive and negative attention. To assess multivariate relationships between brain activity during internal attention and behaviors related to comorbid ADHD, we ran a partial-least squares correlation (PLS-C) analysis in the ADHD_all_ group. After running our primary analysis on how internal attention differed in ADHD compared to HC, we conducted a follow-up subgroup analysis to investigate whether observed differences were driven by ADHD alone rather than heterogeneity stemming from comorbidities. For the follow-up analysis, we selected a subgroup of participants with ADHD who did not have any comorbidities (ADHD_pure_) and excluded patients with ADHD and comorbidities (ADHD_com_). For clarity we refer to patients in our primary analysis as ADHD_all_ (ADHD_all_ = ADHD_pure_ + ADHD_com_).

## Methods

### Participants

We recruited 103 young adults, 55 of whom were patients with ADHD and 48 of whom were HC. Patients were recruited from the Emotion Regulation Disorders Unit at Geneva University Hospitals’ Psychiatry Department; those with existing ADHD diagnoses were not re-diagnosed while the remaining patients were diagnosed with ADHD according to DSM-IV-TR criteria by trained psychologists using the DIGS (Diagnostic Interview for Genetic Studies) or the Kiddie Schedule for Affective Disorders and Schizophrenia (K-SADS). Participants regularly taking medication with a half-life of longer than 24 hours were excluded, and the remaining participants were asked to stop all medication 24 hours before the scan. HC were adults without current psychiatric diagnoses and were matched for age, gender, and educational level. HC were recruited from Geneva and surrounding regions through web announcements. Five participants were excluded from the HC group, two for incomplete scans and three for excessive movement (>3 mm) during the scan. Nine participants with ADHD were excluded (one for incomplete scans, two for excessive movement and six with comorbid ADHD who were taking medications with a half-life of longer than 24 hours). For more details on excluded participants, please refer to Supplemental Materials, Table A. The final analysis was conducted on 89 young adults, 46 of whom were patients with ADHD and 43 of whom were HC. In the follow-up subgroup analysis, we compared 18 patients who had ADHD but without comorbidities (ADHD_pure_) to HC. Demographic data for all participants is shown in Table 1. All participants signed written informed consent in accordance with the approval of the Ethics Committee of the University of Geneva (CER 13-081).

**Table 1.** Demographic Data.

	ADHD_all_ (*N* = 46)	ADHD_pure_ subgroup (*N* = 18)	HC (*N* = 43)
Mean age ± *SD* (years)	23.2 ± 3.5	23.1 ± 3	21.6 ± 3.1
Age range (years)	17–30	20–29	17–29
No. of females	24	12	23
Mean education level ± *SD* (years)	15.2 ± 3.0	15.2 ± 3.3	14.9 ± 3.1
Diagnostic interview	DIGS (44)	DIGS (18)	None
	KSADS (2)		
ADHD presentations	18 combined,	Six combined,	None
	15 predominantly inattentive	Nine predominantly inattentive	
	Five hyperactive-impulsive		
	Eight unspecified	Three unspecified	
Comorbidities	Mood disorders (18)	None	None
	Generalized anxiety disorder (13)		
	Drug abuse (12)		
	Panic attack disorder (11)		
	Social phobia (11)		
	Post-traumatic stress disorder (7)		
	Eating disorders (5)		
	Panic disorders (2)		
	Obsessive-compulsive disorder (2)		
Medications (stopped 24 hours before scan)	Methylphenidate (12)	Methylphenidate (3)	
	Dexmethylphenidate (9)	Dexmethylphenidate (4)	
	Asenapine (1)		
	Trazodone (1)		
	Albuterol (1)	Albuterol (1)	
	Lisdexamfetamine (2)	Lisdexamfetamine (1)	
	Strattera (1)		
	Fluticasone (1)	Fluticasone (1)	

DIGS = diagnostic interview for genetic studies; KSADS = the Kiddie Schedule for Affective Disorders and Schizophrenia (K-SADS).

### Clinical Questionnaires

All participants filled out questionnaires assessing clinical metrics of inattention, hyperactivity, and emotional lability. Inattention and hyperactivity were measured using the World Health Organization’s Adult ADHD Self-Report Scale (ASRS), which has two subscales: ASRS inattention (ASRSi) and ASRS hyperactivity (ASRShi). The ASRS is a reliable measure commonly used in clinical and research settings that is comprised of 18 questions on the recent frequency of ADHD symptoms, taken from the DSM-IV Criterion A for adult ADHD. Emotional lability was assessed using the Affective Lability Scales (ALS) (Harvey et al., 1989). The ALS is a validated, 54-item scale designed to assess self-reported affective changes from a normal mood to other affective states such as depression.

### fMRI Task

The attention task consisted of 112 trials requiring either internal or external attention to stimuli. The stimuli consisted of 28 positively valenced adjectives, 28 negatively valenced adjectives and eight clinically relevant items (e.g., “stressed”). Stimuli were taken from the Profile of Mood State Questionnaire (POMS)(McNair et al., 1971) and each stimulus was presented once during internal trials and once during external trials. Stimuli were comparable for mean word length. The combinations of attention type and valence yielded four task conditions, which assessed differences between internal attention, as measured by internal positive (IntPos) and internal negative (IntNeg) and external attention, as measured by conditions external positive (ExtPos) and external negative (ExtNeg). The four task conditions were presented in a different randomized manner for each participant. This attention task has previously been used in other clinical populations in mood disorder research (Apazoglou et al., 2019).

**Figure 1.**
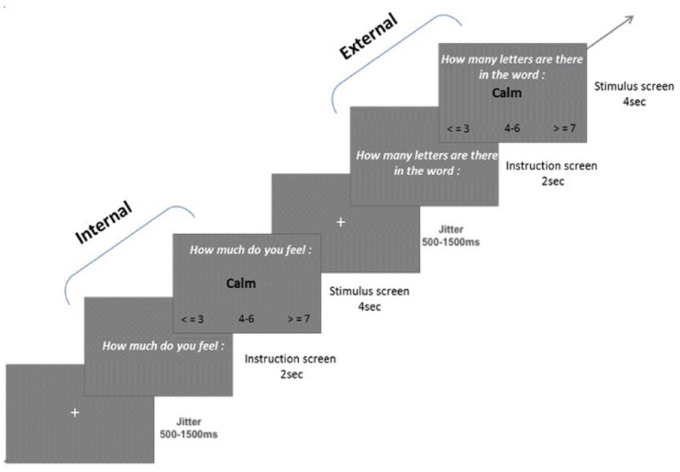
Two trials in the attention task. The first trial demonstrates an example of the internal positive (IntPos) condition while the second demonstrates an external positive (ExtPos) condition.

Each trial began with an instruction screen of 2 seconds stating whether the upcoming stimulus required internal or external attention followed by stimulus presentation for 4 seconds. A numeric scale appeared at the bottom of each stimulus presentation screen that participants used to respond to the task (Figure 1). During Internal trials, participants were asked to evaluate how much they currently felt the state indicated by the word using a numeric scale ranging from ≤3 (meaning they did not feel the state indicated, or they felt it a little bit), to 4−6 (meaning they did feel the state indicated to a moderate extent) to ≥7 (meaning they strongly felt the state indicated) (Joormann et al., 2011). Participants were instructed to respond as quickly as possible. The scale was designed to fit the external condition trials, where participants indicated the number of letters in the word using the same scale. A fixation-cross with a jittered duration between 500 and 1,500 millisecond was shown after each trial and the overall task lasted for a total of 13 minutes. For more details about this paradigm, please refer to Supplemental Materials. This task was implemented using E-Prime 2.0 software (Psychology Software Tools Inc., USA). Reaction times and responses were recorded using an MRI-compatible button box (HH—1 × 4—CR, Current Designs Inc., USA).

### Behavioral Analysis

For the primary analysis comparing the ADHD_all_ group to HC, we first assessed group differences in age, years of education, and questionnaire results using independent sample *T*-tests with Group (ADHD_all_, HC) as the independent variable and age, years of education, and questionnaire results as dependent variables. Group differences in reaction time data were analyzed using mixed model for repeated-measures using Group (ADHD_all_, HC), Attention (Internal, External), and Valence (Positive, Negative) as fixed effects, participant ID as a random variable and reaction time as the dependent variable. The same methodology was used for subgroup analysis except for its Group variable consisted of ADHD_pure_ and HC. Neither the ADHD_all_ group nor the ADHD_pure_ subgroup (Supplemental Materials, Table B) differed in number of invalid trials, as compared to HC. Analyses were conducted using IBM SPSS Statistics, Version 26.0.

### fMRI Acquisition & Processing

#### Acquisition

Functional brain images were acquired with a 3T Magnetom TIM Trio scanner (Siemens, Germany) and a 32-channel head coil using a standard echo-planar imaging sequence [36 transverse slices with 20% gap, 64 × 64 base resolution, voxel size: 3.2 mm ×3.2 mm × 3.2 mm, repetition time (TR): 2,100 millisecond, echo time (TE): 30 millisecond, flip angle (FA): 80°, field of view (FOV): 192 mm]. Anatomical images were also acquired for precise localization and normalization to standard templates, using a T1-weighted 3D sequence (TR/TI/TE: 1900/900/2.32 millisecond, flip angle = 9°, field of view = 230 mm, PAT factor = 2, voxel dimensions: 1 mm, isotropic 256 × 256 × 192 voxel). One run of the attention task was acquired with a total of 380 scans.

#### Preprocessing

Image preprocessing was carried out using standard procedures implemented in SPM12 (http://www.fil.ion.ucl.ac.uk/spm). Functional scans were manually reoriented to place the origin (0, 0, 0) at the anterior commissure and realigned using iterative rigid-body transformations that minimize the residual sum of square between the first and subsequent images and corrected for differences in acquisition time between slices. Participants with a rotation or translation of more than 3 mm (1 voxel) were excluded from further analysis. The structural T1 image was co-registered and normalized with the mean image of the EPI series, and then the data was normalized to the MNI EPI template (2D spline, voxel size: 3 mm) and spatially smoothed with a Gaussian kernel with full width at half maximum (FWHM) of 6 mm.

#### Statistical analysis

First level general linear models of blood oxygen-level dependent (BOLD) variation were modeled for each participant with a design matrix consisting of the four experimental task conditions (IntPos, IntNeg, ExtPos, ExtNeg) which were convolved with a canonical hemodynamic response function. Movement parameters estimated during realignment (*x, y, z* translations and pitch, roll, and yaw rotations) were included as regressors of no interest. In line with previous research using similar experimental paradigms, we analyzed correct trials only (Vetter et al., 2018) to assess group differences when both groups were engaging in the task at hand. There was no significant group difference between the number of incorrect responses during external trials (Supplemental Materials, Table B) and so we discarded the small number of incorrect trials from both groups. Mean framewise displacement as well as ExtPos and ExtNeg trials that were incorrectly answered were modeled as separate regressors of no interest. A high-pass filter with cut-off 128 seconds was applied to remove the low-frequency physiological noise and the default autoregressive AR (1) model was used to estimate residual temporal autocorrelation (Friston et al., 2002). Four contrasts of interest from the weighted beta-images (IntPos IntNeg, ExtPos, & ExtNeg) were then fed into a whole-brain random-effects analysis to measure BOLD variation at the group-level with a 3 × 2× 2 full factorial model. To use the same group-level model for the primary and subgroup analyses, we specified “Group” as the between-subject factor (ADHD_pure_, ADHD_com_, HC) and within-subject factors “Attention” (internal, external) and “Valence” (positive, negative). Importantly, during the primary analysis, we treated ADHD_pure_ and ADHD_com_ as one group (ADHD_all_ = ADHD_pure_ + ADHD_com_). Two manipulation checks assessing the main effect of attention (Internal > External and External > Internal) were conducted across all participants to verify the validity of the fMRI attention task (Supplemental Materials, Table C). We also assessed main effect of Group (ADHD_all_ > HC and HC > ADHD_all_) (Supplemental Materials, Table D). Importantly, we performed statistical comparisons between groups during internal and external attention as well as during the four individual task conditions as planned comparisons of simple effects. Whole-brain results for the primary analysis, ADHD_all_ compared to HC, are reported at a cluster-level threshold of *p*-FWE < .05. Brain regions were identified using the Harvard-Oxford atlas distributed with FSL (http://www.fmrib.ox.ac.uk/fsl/).

The methodology for the subgroup analysis was identical to that of the primary analysis. The same full factorial model created in the primary analysis was used to assess differences in brain activations between ADHD_pure_ and HC. Results are reported at a corrected cluster-level threshold of *p*-FWE < .05 and with a cluster-forming threshold of voxel-level *p* < .001.

### Partial Least Squares Correlation

To assess multivariate relationships between behavior and brain activations during internal and external attention, we ran a partial least squares correlation (PLS-C) analysis (Krishnan et al., 2011) in the ADHD_all_ group. PLS-C is a well-established methodology used to assess brain-behavior relationships in various clinical populations (Delavari et al., 2022; Ziegler et al., 2013; Zöller et al., 2017) and we employed it using myPLS, a publicly available, Matlab-based toolbox (https://github.com/MIPLabCH/myPLS). To summarize the methodology used in PLS-C, we first computed correlations between matrix *Y*, which consisted of participants’ behavioral scores (ASRSi, ASRShi, and ALS), and matrix *X*, which consisted of voxel data per subject during the four task conditions (IntPos, IntNeg, ExtPos, ExtNeg). The resulting correlation matrices were concatenated into a common correlation matrix, *R* = X^T^Y. Matrix *R* then underwent singular value decomposition, resulting in latent variables or correlation components. Each correlation component is a combination of brain activations and behavior weights, which indicate how strongly each variable contributes to the multivariate brain-behavior correlation. These values can be interpreted similarly to correlation values. Significance of correlation components was determined by permutation testing (1,000 permutations) and the stability of brain and behavior weights was ensured using bootstrapping (500 bootstrap samples). For more details on singular value decomposition and correlation components, please refer to previous publications using the myPLS toolbox (Zöller et al., 2017).

## Results

### Behavioral and Clinical Data

The primary analysis revealed no group difference between ADHD_all_ and HC in average reaction times, nor age, gender, or education level. Regarding clinical measures of inattentiveness, the ADHD_all_ group (mean = 22.8 ± 7.2) scored significantly higher (*t* = 7, *df* = 83, *p* < .001) than HC (13.2 ± 5.6). For hyperactivity, ADHD_all_ (mean = 17.5 ± 7.7) also scored significantly higher (*t* = 5.4, *df* = 83, *p* < .001) than HC (9.9 ± 5.6). Finally, ADHD_all_ (mean = 1.1 ± 0.6) also scored significantly higher (*t* = 4.5, *df* = 83, *p* < .001) than HC (0.6 ± 0.3) in affective lability.

Subgroup analysis revealed no differences between the ADHD_pure_ subgroup and HC in reaction times, age, sex, nor educational level. The ADHD_pure_ subgroup scored higher on inattentiveness (ADHD_pure_ = 24 ± 7.1, HC = 13.2 ±5.6, *t* = 5.7, *df* = 26, *p* < .001) and on hyperactivity compared to HC (ADHD_pure_ = 17.1 ± 7.6, HC = 9.9 ± 5.6, *t* = 3.6, *df* = 25, *p* < .001). No other differences were found.

### fMRI Results

#### Manipulation checks

To verify task validity, we ran two manipulation checks. The first contrasted Internal Attention > External Attention and revealed increased activations in regions including the inferior frontal gyrus, orbitofrontal cortex, and angular gyrus (Supplemental Materials, Table C). The second manipulation check contrasted External Attention > Internal Attention and revealed increased activation in regions including the occipital cortex, striatum, middle frontal gyrus, and thalamus (Supplemental Materials, Table C). Interaction contrasts between Group (ADHD_all_ > HC and HC >ADHD_all_) and Attention (Internal Attention > External Attention and External Attention > Internal Attention) revealed no significant effects.

#### Main effects of group, ADHD_all_ versus HC

We assessed main effects of group, across all conditions (IntPos, IntNeg, ExtPos, and ExtNeg). Results revealed that the ADHD_all_ group, as compared to HC, had increased activations in regions such as the angular gyrus and occipital fusiform gyrus (Supplemental Materials, Table D). The ADHD_all_ group, as compared to HC, had diminished activation in regions including the insular cortex, putamen, and precentral gyrus (Supplemental Material, Table D).

#### Group differences during internal and external attention

**Figure 2.**
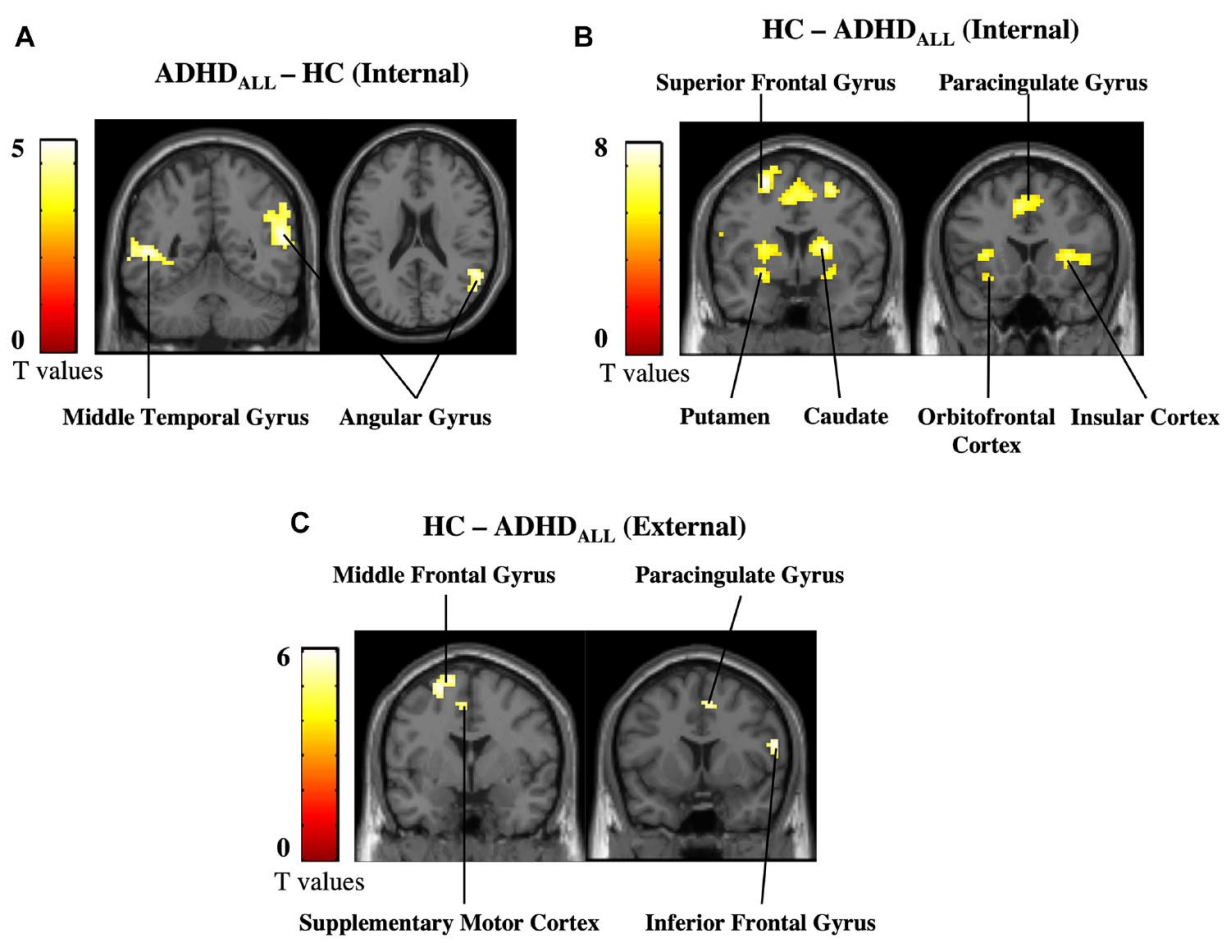
Primary analysis results. (a) Increases in activation in ADHD_all_ compared to HC during internal attention, (b) decreases in activation in ADHDall compared to HC during internal attention, and (c) decreases in activation in ADHD_all_ compared to HC during external attention. Results are shown at a cluster-level threshold of *p*-FWE < .05.

Next, we assessed simple effects of group during internal (IntPos + IntNeg trials) and external (ExtPos + ExtNeg trials) attention, as planned comparisons. Results for internal attention trials showed that, as compared to HC, the ADHD_all_ group had increased activity in the angular gyrus and middle temporal gyrus and diminished activity in the insular cortex and the fronto-striatal circuity, including the putamen, caudate, and orbitofrontal cortex (Figure 2; Table 2). During external attention trials, the ADHD_all_ group had increased activity in the occipital cortex and diminished activity in regions including the superior frontal gyrus, supplementary motor cortex and paracingulate cortex (Figure 2c; Supplemental Material, Table E). No other significant effects were found.

**Table 2.** Group Differences During Internal Trials.

Contrast	Region	*p*	Cluster size	*T*	MNI coordinates		
					*x*	*y*	*z*
ADHD_all_ - HC (Internal)	Angular gyrus	0	229	4.47	54	−51	21
				4.25	57	−60	15
				4.13	48	−57	33
	Middle temporal gyrus	.029	92	4.36	−48	−51	6
				3.81	−60	−51	3
				3.41	−33	−51	−3
HC - ADHD_all_ (Internal)	Superior frontal gyrus	0	327	7.57	−24	0	60
				6.17	−15	0	69
				5.71	−36	−18	60
	Paracingulate gyrus	0	283	7.54	9	9	48
				6.42	−6	15	45
				6.23	−3	0	51
	Putamen	0	305	7.32	21	0	12
				7.16	57	9	18
				6.44	33	21	6
	Middle frontal gyrus	0	43	6.69	27	3	54
	Lateral occipital cortex	0	69	6.42	−18	−72	51
		0	174	6.2	−24	3	12
				6.12	−33	18	6
				6.07	−27	3	−9
	Lateral occipital cortex	0	23	5.89	18	−63	60
	Frontal pole/inferior parietal gyrus	0	25	5.79	−42	45	6
				5.02	−45	39	15
	Orbito frontal cortex	.003	10	5.59	−30	18	−12
	Precentral gyrus/inferior parietal gyrus	.009	5	5.41	−57	6	21
	Putamen	.001	20	5.34	24	6	−12
	Paracingulate gyrus	.001	18	5.29	12	24	33
	Occipital cortex	.006	7	5.16	42	−81	6

*Note. p*-FWE < 0.05 at the cluster-level.

The same simple effects analysis was conducted for ADHD_pure_ subgroup analysis. During internal attention, the ADHD_pure_ subgroup had increased activation in a cluster in the angular gyrus compared to HC (*x* = 30, *y* = −42, *z* = 42, kE = 165, *T* = 5.34, *p* = .0002). No other significant group differences were found.

#### Group differences during task conditions

**Figure 3.**
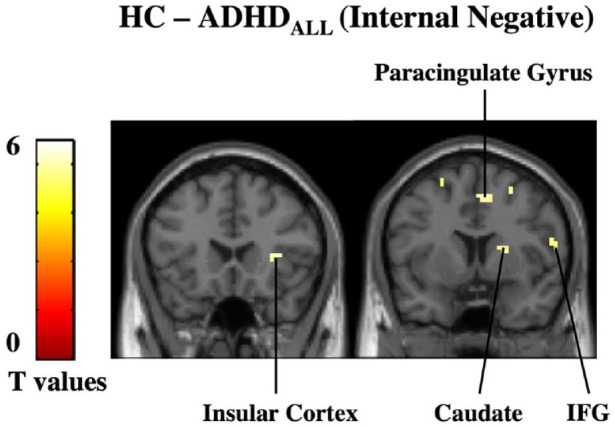
Group differences during IntNeg trials. (a) During negatively-valenced, internal attention trials, the ADHD_all_ subgroup had diminished activation in the insular cortex, caudate, paracingulate gyrus, and inferior frontal gyrus (IFG) compared to HC. Results are reported at a corrected cluster-level threshold of *p*-FWE < .05.

Next, we assessed whether group differences during internal attention and external attention were modulated by valence by looking within the four task conditions (IntPos, IntNeg, ExtPos, and ExtNeg, respectively). Results revealed that during IntPos trials the ADHD_all_ group had diminished activation in the bilateral putamen, paracingulate gyrus and superior frontal gyrus compared to HC. During IntNeg trials, the ADHD_all_ group had diminished activation in regions including the insular cortex, paracingulate gyrus, caudate, and IFG (Figure 3; Table 3). No other significant results were found. No significant results were found in the ADHD_pure_ subgroup analysis.

**Table 3.** Group Differences During Individual Trial Conditions.

Contrast	Region	*p*	Cluster size	*T*	MNI coordinates		
					*x*	*y*	*z*
HC - ADHD_all_ (Internal positive)	Paracingulate gyrus	0	28	5.64	9	9	48
	Superior frontal gyrus	0	26	5.56	−21	3	60
				4.87	−15	0	69
	Putamen	0	31	5.5	21	0	12
	Paracingulate gyrus	.008	6	4.9	−6	15	42
	Putamen	.006	7	4.88	−24	3	12
HC - ADHD_all_ (Internal negative)	Superior frontal gyrus	0	27	5.66	−24	0	60
	Precentral gyrus/inferior frontal gyrus	.001	20	5.55	57	9	18
	Paracingulate gyrus/ supplementary motor cortex	0	24	5.53	6	9	51
	Putamen	.001	18	5.31	21	0	12
	Middle frontal gyrus	.008	6	5.25	27	3	54
	Insular cortex	.004	9	5.1	33	21	6
	Occipital cortex	.005	8	5.01	−15	−72	51

*Note. p*-FWE < 0.05 at the cluster-level.

#### Partial least squares correlation

**Figure 4.**
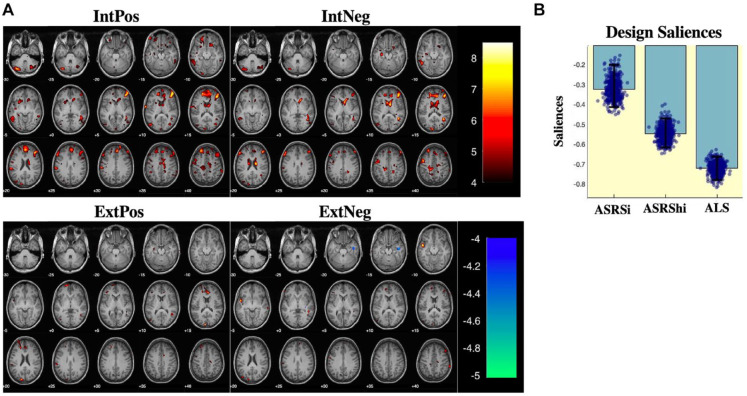
Partial least squares - correlation results. (a) Multivariate patterns reveal that the comorbid ADHD group had increased activations in the orbitofrontal cortex, striatum, and frontal gyri, including the inferior frontal gyrus (IFG), during IntPos and IntNeg trials but not during ExtPos and ExtNeg trials. (b) Design saliences which show the loadings of our three behaviors of interest on the brain data. The pattern of neural activation was most strongly negatively correlated with affective lability (as measured by ALS scores), and moderately negatively correlated with ADHD symptomology (as measured by ASRSi and ASRShi scores).

PLS-C analysis resulted in one significant latent component (*p* < .05, *r* = .59) that captured brain saliences representing voxels strongly correlated with affective lability and ADHD symptoms during IntPos and IntNeg trials but not during ExtPos and ExtNeg trials (Figure 4a). Due to the high ALS loading, the corresponding pattern of brain salience bootstrap scores (Figure 4b) contains the voxels where BOLD activation is strongly correlated with affective lability, moderately correlated to hyperactivity and weakly correlated to inattention in patients with ADHD. During internal attention, a similar pattern of activity is seen across IntPos and IntNeg trials in that the less affectively labile and symptomatic participants with ADHD were, the more activation they had in the orbitofrontal cortex, striatum, inferior frontal gyrus, supramarginal gyrus, and cerebellum.

## Discussion

The present study assessed how the neural bases of internally oriented attention differed in young adults with ADHD compared to controls, and how positive and negative valence modulated these processes. To this end, we employed a word processing fMRI task during which participants alternated between paying internal and external attention. This study revealed distinct patterns of brain activations associated with internal attention in our ADHD group, such that they have more activity in regions associated with mind-wandering and information integration and diminished activations in subcortical regions associated with self- and emotion-processing compared to controls.

When prompted to reflect upon internal states, the ADHD_all_ group had increased activation in the right angular gyrus (rAG) compared to controls. A recent meta-analysis on this region in the lateral parietal cortex characterized it as a dynamic, online buffer involved in combining internal and external information (Humphreys et al., 2021). Humphreys and colleagues state the rAG helps combine autobiographical experiences and conceptual knowledge structures in a manner necessary for higher-order, cognitive functions such as constructing internal models of the world (Hasson et al., 2008) or reconstructing autobiographical memories (Lerner et al., 2011). In the present study, increased rAG activation during internal attention may suggest it is more costly for ADHD populations to converge external information (such as the experimental setting and task at hand) with internal information (current internal state). It is possible that the information processing style in ADHD is less fluid than in controls, such that they get stuck in semantic and cognitive processing instead of integrating interoceptive information and activation brain regions involved in emotion regulation. Our results further indicate this difficulty is a property of ADHD itself; subgroup analyses revealed ADHD_pure_ compared to controls also had increased activation of right rAG during internal attention suggesting that pure ADHD was driving increased rAG activation.

In terms of diminished activations during internal attention, the ADHD_all_ group had decreased activity in self- and emotion-processing regions such as the insula, inferior frontal gyrus, and striatum compared to controls. Looking within individual task conditions revealed a similar pattern of activation during IntNeg trials suggesting that these activations were driven by a combination of internally oriented and negatively valenced emotional attention. These finding are reasonable given that the relationship between emotion and attention is intertwined, with the saliency needed for a stimulus to emerge from environmental noise being directly related to its emotional characteristics in terms of valence and arousal (Janak & Tye, 2015). Diminished activation during internal attention of the insula, which serves an important role in attention and converging internal bodily states to emotional experiences (Zaki et al., 2012), is consistent with the notion of adults with ADHD having a decreased capacity to reflect upon their internal state compared to controls (Perroud et al., 2017).

Interestingly, within internal attention trials, ADHD_all_ showed diminished activation in the bilateral putamen during IntPos trials in compared to controls. This is in line with previous research showing a reduced capacity to process positive emotions in ADHD (Conzelmann et al., 2009; Ibáñez et al., 2011), which may reflect differences in ADHD populations in terms of reward processing and motivation (Scheres et al., 2007; Sonuga-Barke et al., 2008). We also found decreased activation in the bilateral paracingulate cortex and going into the pre-supplementary motor area. The pre-supplementary motor area helps link the onset of movements with motivations (Rizzolatti & Luppino, 2001) and both animal models (Luppino et al., 1993) and human (Krolak-Salmon et al., 2006; Osaka et al., 2003) research have implicated it in processing positive emotions such as happiness.

The findings of the simple effects of group during IntPos and IntNeg trials help to contextualize the results of the PLS-C analysis in comorbid ADHD. The multivariate analysis revealed that a similar pattern of diminished activity seen in comorbid ADHD compared to HC during internal attention, correlated with increased affective lability and ADHD symptomology within the comorbid ADHD group. This result is notable for several reasons, the first being that, like the simple effects of group during internal and external attention, diminished activation in the frontal cortex and the striatum was specific to internal attention. Secondly, the PLS-C analysis revealed that behavior most correlated with brain activations was affective lability, not ADHD symptomology. Given that emotional dysregulation is increasingly seen as a core symptom of ADHD, this finding is not contrary to existing literature. More importantly however, it suggests that in comorbid ADHD such as the participants in the present study, emotional dysregulation comes to the forefront of ADHD symptoms.

During external attention trials we found diminished activation in the superior frontal gyrus, the bilateral paracingulate cortex, and the operculum cortex. Previous research generally shows hyperresponsiveness toward emotional distractors in ADHD (controlling for other Axis I disorders) compared to controls: one study showed insula and inferior frontal gyrus hyperresponsiveness toward negatively valenced distractors (Vetter et al., 2018), while others showed that emotional distractors are associated with increased functional connectivity between the amygdala and emotion processing hubs (Posner et al., 2011) as well as striatal and occipital regions in ADHD compared to controls (Hwang et al., 2015). Other group differences seen during external attention include altered activation patterns in visual and motor planning cortices, which are largely in line with previous research (Brace et al., 2015; Lenz et al., 2010).

Our results should be contextualized regarding the study’s limitations. First, our fMRI paradigm did not include a neutral valence condition, so we were not able to detect differences stemming from positive versus neutral and negative versus neutral stimuli. Second, we underline the preliminary nature of the subgroup analysis, given the small size of the pure ADHD subgroup. Finally, subject head motion is a well-known, major source of noise in fMRI; despite controlling for rotation and translation, as well as mean framewise displacement, it is possible that our results are influenced by noise related to movement (Friston et al., 1996).

The present study underlines the importance of studying effects of internal attention and emotion processes affected in ADHD, and suggests these processes involve altered rAG functioning in ADHD. In line with this, future studies with larger sample sizes of pure ADHD on internal emotional attention are needed to replicate the present results. More broadly, future fMRI studies researching if and how internal attention relates to executive dysfunction and emotion regulation capacities in ADHD populations would build upon current results and shed light on our current understanding of internal attention and emotion.

## Supplemental Material

sj-docx-1-jad-10.1177_10870547221147546 – Supplemental material for Neural Basis of Internal Attention in Adults with Pure and Comorbid ADHD Supplemental material, sj-docx-1-jad-10.1177_10870547221147546 for Neural Basis of Internal Attention in Adults with Pure and Comorbid ADHD by Halima Rafi, Ryan Murray, Farnaz Delavari, Nader Perroud, Patrik Vuilleumier, Martin Debbané and Camille Piguet in Journal of Attention Disorders
