# Supplementary material for: Neural Basis of Internal Attention in Adults with Pure and Comorbid ADHD
Source: J Atten Disord. 2023 Jan 12;27(4):423–36. doi: 10.1177/10870547221147546 (PMC13035225; doi:10.1177/10870547221147546)
Supplement: sj-docx-1-jad-10.1177_10870547221147546 – Supplemental material for Neural Basis of Internal Attention in Adults with Pure and Comorbid ADHD [file sj-docx-1-jad-10.1177_10870547221147546.docx]

Supplementary Materials

Additional Information about the fMRI Paradigm

The attention task consisted of two attention orientations (internal or external) and two stimuli valences (positive or negative). This resulted in 4 task conditions (IntPos, IntNeg, ExtPos and ExtNeg), as shown in the 4x4 table below:

|  | **Internal Attention** | **External Attention** |
| --- | --- | --- |
| **Positive Valence** | IntPos | ExtPos |
| **Negative Valence** | IntNeg | ExtNeg |

Examples of positive and negative stimuli (in French) used in this paradigm are shown in the table below:

| Positive Stimuli | Negative Stimuli | |
| --- | --- | --- |
| Calme | Agité |  |
| Attentif | Confus |  |
| Joyeux | Triste |  |
| Amical | Découragé |  |
| Confiant | Anxieux |  |

Table A. Demographic Information for Excluded Participants

|  | ADHD_all_  (*N* = 9) | HC  (*N* = 5) |
| --- | --- | --- |
| Mean Age ± SD (years) | 23.1 ± 4.7 | 21.2 ± 6.5 |
| Age Range | 17-30 | 17-31 |
| No. of Females | 4 | 4 |
| Mean Education Level ± SD (years) | 14.4 ± 3.5 | 13 ± 3.2 |
| ADHD Presentations | 3 Combined, 2 Inattentive  2 Hyperactive-Impulsive, 2 unspecified | None |
| Comorbidities | Mood disorders (1)  Generalized anxiety disorder (3)  Panic attack disorder (3)  Social phobia (2)  Agoraphobia (1)  Post-traumatic stress disorder (1)  Eating disorder (1) | None |
| ASRSi | 22.5 ± 6.8 | 9 ± 4.6 |
| ASRShi | 20.1 ± 8.5 | 5 ± 4.6 |
| ALS | 1.6 ± 0.5 | 0.6 ± 0.4 |

All participants with ADHD had comorbidities. Independent sample t-tests revealed no difference (p<0.05) between included and excluded participants in terms of mean age, education level nor clinical scores of inattention, hyperactivity nor affective lability (*p*>0.05).

Table B. Independent Sample T-tests assessing the No. of Invalid Trials per Group

| Mean No. of Invalid Trials ± Standard Deviation | | | Ind. Sample T-test,  ADHD_all_ compared to HC | | | Ind. Sample T-test,  ADHD_pure_ compared to HC | | |
| --- | --- | --- | --- | --- | --- | --- | --- | --- |
| ADHD_all_  (*N* = 46) | ADHD_pure_  (*N* = 18) | HC  (*N* = 43) | df | *F*-Statistic | *p* | df | *F*-Statistic | *p* |
| 7.8 ± 6.7 | 7.9 ± 5.9 | 6.7± 3.8 | 87 | 5.8 | 0.50 | 59 | 0.49 | 0.41 |

### Table C. Manipulation Checks

| Contrast | Region | *p* | Cluster Size | T | MNI Coordinates | | |
| --- | --- | --- | --- | --- | --- | --- | --- |
|  |  |  |  |  | x | y | z |
| External - Internal | Putamen | 0 | 381 | 8.61 | 21 | 12 | 0 |
|  |  |  |  | 8.44 | 21 | 9 | -9 |
|  | Putamen | 0 | 334 | 7.91 | -21 | 6 | 6 |
|  |  |  |  | 5.07 | -15 | -15 | 12 |
|  | Lateral Occipital Cortex / Inferior Temporal Gyrus | 0 | 329 | 6.5 | -48 | -63 | -9 |
|  |  |  |  | 6.26 | -30 | -90 | -6 |
|  |  |  |  | 5.78 | -33 | -87 | 12 |
|  | Occipital Pole | 0 | 135 | 5.81 | 24 | -90 | -3 |
|  |  |  |  | 5.53 | 15 | -87 | 0 |
|  |  |  |  | 5.12 | 36 | -81 | 6 |
|  | Cerebellum | 0 | 56 | 5.65 | -3 | -75 | -30 |
|  | Caudate | 0.001 | 22 | 5.41 | -21 | 30 | 3 |
|  | Inferior Temporal Gyrus | 0.001 | 18 | 5.41 | 51 | -60 | -12 |
|  | Precentral Gyrus | 0 | 25 | 5.39 | 42 | 3 | 24 |
|  | Thalamus | 0 | 24 | 5.24 | 12 | -15 | 9 |
|  | Cerebellum | 0.009 | 5 | 5.02 | -24 | -66 | -45 |
|  | Cerebellum | 0.008 | 6 | 4.99 | 27 | -60 | -24 |
|  | Middle Frontal Gyrus/  Precentral Gyrus | 0.009 | 5 | 4.9 | 33 | -3 | 48 |
|  | Occipital Pole | 0.006 | 7 | 4.83 | 30 | -69 | 30 |
|  | Cerebellum | 0.009 | 5 | 4.82 | -24 | -66 | -21 |
| Internal - External | Inferior Frontal Gyrus | 0 | 276 | 9.88 | -51 | 27 | 3 |
|  | OrbitoFrontal Cortex |  |  | 8.63 | -42 | 27 | -15 |
|  | Occipital Cortex/Angular Gyrus | 0 | 56 | 5.57 | -48 | -63 | 24 |
|  | Middle Temporal Gyrus | 0.001 | 15 | 5.42 | -54 | -6 | -18 |
|  | Inferior Frontal Gyrus | 0.005 | 8 | 5.31 | 54 | 30 | 3 |
|  | Superior Frontal Gyrus | 0.006 | 7 | 5.14 | -9 | 54 | 21 |

*p*<.05 FWE-corrected at the cluster-level

Table D. Main Effect of Group (across all trials conditions)

| Contrast | Region | *p* | Cluster Size | T | MNI Coordinates | | |
| --- | --- | --- | --- | --- | --- | --- | --- |
|  |  |  |  |  | x | y | z |
| ADHD_all_ - HC | Lingual Gyrus | 0.002 | 12 | 6.18 | 12 | -87 | -12 |
|  | Occipital Fusiform Gyrus | 0 | 37 | 5.77 | -18 | -81 | -15 |
|  |  | 0.002 | 13 | 5.67 | 30 | 6 | 27 |
|  | Superior Temporal Gyrus | 0.004 | 9 | 5.47 | -60 | -27 | 0 |
|  | Angular Gyrus | 0.001 | 21 | 5.38 | 54 | -51 | 18 |
|  | Middle Temporal Gyrus | 0.001 | 17 | 5.08 | -48 | -51 | 6 |
|  |  |  |  | 4.97 | -36 | -51 | 0 |
|  | Hippocampus | 0.009 | 5 | 4.9 | 21 | -39 | 9 |
| HC - ADHD_all_ | Superior Frontal Gyrus | 0 | 1168 | 8.85 | -24 | 0 | 63 |
|  |  |  |  | 8.54 | 9 | 9 | 48 |
|  |  |  |  | 7.77 | -6 | 15 | 45 |
|  | Precentral Gyrus/ Inferior Frontal Gyrus | 0 | 352 | 8.43 | 57 | 9 | 18 |
|  |  |  |  | 7.35 | 21 | 0 | 12 |
|  |  |  |  | 6.84 | 33 | 21 | 6 |
|  | Middle Frontal Gyrus | 0 | 81 | 7.36 | 27 | 3 | 54 |
|  | Lateral Occipital Cortex | 0 | 57 | 7.18 | 18 | -63 | 60 |
|  |  |  |  | 5.86 | 27 | -78 | 48 |
|  | Lateral Occipital Cortex | 0 | 104 | 7.02 | -18 | -72 | 51 |
|  |  |  |  | 6.64 | -18 | -66 | 60 |
|  | Insular Cortex | 0 | 339 | 6.86 | -33 | 18 | 6 |
|  |  |  |  | 6.69 | -57 | 6 | 21 |
|  |  |  |  | 6.61 | -24 | 3 | 12 |
|  | Orbitofrontal Cortex / Insular Cortex | 0 | 31 | 6.8 | -30 | 18 | -12 |
|  | Frontal Pole | 0 | 71 | 6.79 | -42 | 45 | 6 |
|  |  |  |  | 6.65 | -45 | 39 | 15 |
|  | Lateral Occipital Cortex | 0.001 | 20 | 6.15 | 45 | -81 | 3 |
|  | Operculum Cortex | 0 | 57 | 5.7 | -45 | -27 | 18 |
|  |  |  |  | 5.69 | -33 | -30 | 21 |
|  |  |  |  | 5.64 | -57 | -18 | 18 |
|  | Postcentral Gyrus | 0.003 | 11 | 5.69 | 45 | -27 | 45 |
|  | Putamen | 0.001 | 16 | 5.29 | -27 | 3 | -9 |
|  |  |  |  | 5.29 | -21 | 0 | -15 |
|  | Brainstem | 0.003 | 11 | 5.23 | -6 | -21 | -24 |
|  |  |  |  | 4.81 | 9 | -18 | -24 |
|  | Cerebellum | 0.003 | 10 | 5.09 | 45 | -57 | -33 |

*p*<.05 FWE-corrected at the cluster level

Table E. Simple Effect of Group during External Attention

| Contrast | Region | *p* | Cluster Size | T | MNI Coordinates | | |
| --- | --- | --- | --- | --- | --- | --- | --- |
|  |  |  |  |  | x | y | z |
| ADHD_all_ - HC (External) | Lingual Gyrus | 0.006 | 7 | 5.43 | 12 | -87 | -12 |
|  | Occipital Fusiform Gyrus | 0.005 | 8 | 4.86 | -18 | -84 | -18 |
| HC - ADHD_all_ (External) | Superior Frontal Gyrus | 0 | 165 | 6.03 | -24 | 0 | 63 |
|  |  |  |  | 5.66 | -15 | 0 | 69 |
|  |  |  |  | 5.27 | -45 | -30 | 48 |
|  | Precentral gyrus | 0 | 26 | 5.72 | 57 | 9 | 18 |
|  | Paracingulate / Supplementary Motor Cortex | 0.001 | 18 | 5.51 | 9 | 9 | 48 |
|  | Paracingulate | 0 | 26 | 5.45 | -6 | 15 | 42 |
|  | Frontal Pole /  Inferior Parietal Gyrus | 0.008 | 6 | 5.25 | -45 | 36 | 15 |
|  | Lateral Occipital Cortex | 0.006 | 7 | 5.12 | 18 | -63 | 63 |
|  | Supplementary Motor Cortex | 0.004 | 9 | 5.11 | -6 | -3 | 51 |
|  | Operculum Cortex | 0.009 | 5 | 4.72 | -45 | -24 | 18 |

*p*-FWE<.05 at the cluster-level
